# Pertussis in infants: an underestimated disease

**DOI:** 10.1186/s12879-016-1710-0

**Published:** 2016-08-15

**Authors:** Anna Chiara Vittucci, Valentina Spuri Vennarucci, Annalisa Grandin, Cristina Russo, Laura Lancella, Albero Eugenio Tozzi, Andrea Bartuli, Alberto Villani

**Affiliations:** 1 General Pediatric and Infectious Diseases Unit, Bambino Gesù Children’s Hospital, IRCCS, Rome, Italy; 2 Virology Unit, Bambino Gesù Children’s Hospital, IRCCS, Rome, Italy; 3 Epidemiology Unit, Bambino Gesù Children’s Hospital, IRCCS, Rome, Italy; 4 Rare Disease and Medical Genetics Unit, Bambino Gesù Children’s Hospital, IRCCS, Rome, Italy

**Keywords:** Bordetella *pertussis*, Infants, Resurgence, Respiratory symptoms, Real time polymerase chain reaction

## Abstract

**Background:**

The clinical diagnosis of pertussis is not easy in early infancy since clinical manifestations can overlap with several different diseases. Many cases are often misclassified and underdiagnosed. We conducted a retrospective study on infants to assess how often physicians suspected pertussis and the actual frequency of Bordetella *pertussis* infections.

**Methods:**

We analyzed all infants with age ≤90 days hospitalized from March 2011 until September 2013 for acute respiratory symptoms tested with a Real Time Polymerase Chain Reaction able to detect Bordetella *pertussis* and with a Real Time Polymerase Chain Reaction for a multipanel respiratory virus. Therefore, we compared patients with pertussis positive aspirate, patients with respiratory virus positive aspirate and patients with negative aspirate to identify symptoms or clinical findings predictive of pertussis.

**Results:**

Out of 215 patients analyzed, 53 were positive for pertussis (24.7 %), 119 were positive for respiratory virus (55.3 %) and 43 had a negative aspirate (20 %). Pertussis was suspected in 22 patients at admission and 16 of them were confirmed by laboratory tests, while 37 infants with different admission diagnosis resulted positive for pertussis. The sensitivity of clinical diagnosis was 30.2 % and the specificity 96.3 %. Infants with pertussis had more often paroxysmal cough, absence of fever and a higher absolute lymphocyte count than infants without pertussis.

**Conclusions:**

Pertussis is a serious disease in infants and it is often unrecognized; some features should help pediatricians to suspect pertussis, but clinical suspicion has a low sensitivity. We suggest a systematic use of Real Time Polymerase Chain Reaction to support the clinical suspicion of pertussis in patients with less than 3 months of age hospitalized with acute respiratory symptoms.

## Background

Despite a widespread vaccination program, pertussis continues to be a common worldwide infection in pediatric and adult populations. In the past decade, there has been a resurgence of this disease in United States and European countries, peaking every 2 to 5 years [1–7]. The last peak reported in Europe was in 2012 [8].

In contrast to what is reported in other countries, in Italy after the introduction of acellular vaccine in 1995 incidence has continued to decrease and pertussis has not reemerged yet [9, 10]. Therefore epidemic cycles have been clearly less identifiable due to the low incidence [10]. In our country, vaccination schedule provides a pertussis vaccine dose at 3, 5 and 12 months and a booster is recommended in the preschool period and in adolescents. Vaccination coverage during the analyzed period was around 95 % [11].

Since other countries with high immunization coverage over a long period of time experienced a resurgence of pertussis [3, 5], we hypothesized that the epidemiology of this disease in Italy may be affected by the lack of recognition by clinicians with the consequence of limiting the use of laboratory confirmation [9]. In clinical practice the diagnosis of pertussis is generally reached without microbiological confirmation leading to a possible lack of clinical awareness to start early treatment and prevent complications.

Infants are known to acquire pertussis from adolescents’ and adults’ contacts that return susceptible to the disease because of waning immunity as well as from unvaccinated children [12–14]. Clinical manifestations may be different depending on age. Severe symptoms are common in young unvaccinated infants and pertussis continues to be a major cause of vaccine-preventable death in this age group [15]. However, cases with atypical clinical presentations do occur and may be often unrecognized, especially during the winter season, when other respiratory viruses circulate and the minimum incidence of pertussis is usually observed. The annual seasonality in the Italian pertussis incidence peaked between March and August while the minimum incidence has been observed between September and February [10].

We therefore systematically studied a series of infants ≤3 months of age hospitalized with respiratory symptoms to detect how frequently physicians suspected pertussis on a clinical basis and the actual frequency of laboratory confirmed cases. We compared patients with pertussis infections and patients with other respiratory infections to identify clinical and laboratory predictors of pertussis.

## Methods

The study involved patients with age ≤3 months admitted at Pediatric Department of Bambino Gesù Children’s Hospital in Rome from March 1st, 2011 until September 30th, 2013, with acute respiratory symptoms or conditions (cough, dyspnea, rhinorrhea, bronchiolitis, apnea, acute life threatening episode). For all the patients, a nasopharyngeal specimen was tested for Bordetella *pertussis* (BP) and for viruses generally associated to respiratory infections (RV) such as Adenovirus, Influenzae Virus, Parainfluenzae Virus, Respiratory Syncytial Virus (RSV), Metapneumovirus, Coronavirus and Rhinovirus. The samples were obtained within 24 h of admission by trained nurses and processed within 48–72 h.

The medical records of subjects were retrospectively reviewed. Information collected included age, gender, medical history, immunization status, clinical presentation, ongoing antibiotic therapy, admission diagnosis, length of illness, length of hospital stay, laboratory test results, concurrent infections and complications (requirement of oxygen therapy, pneumonia, death).

Patients who had received the first dose of DPT vaccine were excluded from the study.

For nucleic acid extraction 10 μl of internal control (IC) were added to 200 μl of sample for BP and to 400 μl for RV, obtaining 90 and 60 μl of eluate respectively, by using the automatic “magnetic beads-based” EZ1 Advanced XL (Qiagen) instrument.

Primers and Probe for IC were provided by the manufacturer (Argene, Biomerieux, Marcy l’Etoile, France). The IC in use for this assay is referring to a “capsided” synthetic sequence that need to be lysed before being processed in extraction protocol and handled as well as “regular” specimen.

The bacterial DNA was processed immediately on Taqman platform (Applied Biosystem) with Bordetella R-gene™ assay (Argene, Biomerieux, Marcy l’Etoile, France), able to amplify a fragment of 191 bp of the target region IS481 for BP. The ready-to-use amplification mixture included primers, dNTPs, amplification buffer, Taq Polymerase, probes specific for Bordetella and for the IC. The target DNA was amplified through the Taq Polymerase activation at 95 °C for 15 min, and 45 repeats of denaturation at 95 °C for 10 s and hybridization/elongation at 60 °C for 40 s.

The Real Time-Polymerase Chain Reaction (RT-PCR) targeting IS481 is the most commonly used diagnostic tool when suspecting pertussis given its high sensitivity since up to 238 copies of IS481 is found in the BP genome. The high sensitivity does not correspond to high specificity, as few copies of IS481 are also present in the genome of Bordetella *holmesii* and Bordetella *bronchiseptica* [16].

We assumed as positive patient for pertussis a subject who received a positive RT-PCR result (following described as BP+).

For RV detection samples were analyzed by reverse transcription and gene amplification with RT-PCR.

All procedures performed in studies were in accordance with the ethical standards of the institutional and national research committee and with the 1964 Helsinki declaration and its later amendments or comparable ethical standards. The study was approved by the Institutional Scientific Review Board of Bambino Gesù Children’s Hospital.

### Statistical analysis

We described the characteristics of patients, their symptoms and clinical findings. Patients were divided into three groups: patients with BP positive aspirate (BP+), patients with RV positive aspirate (RV+) and patients with BP and RV negative aspirate (BP-RV-). Comparisons across groups were performed through ANOVA for continuous measures and chi-square for discrete variables. To assess the symptoms or clinical findings predictive of pertussis we applied a logistic regression model in which the dependent variable was dichotomous (pertussis yes/no).

We also calculated the specificity and sensitivity of clinical suspicion of pertussis using as gold standard the result of RT PCR for pertussis on nasopharyngeal aspirate.

## Results

During the study period, we enrolled 215 patients. The admission diagnosis of those patients is reported in Table 1.

**Table 1:** Diagnosis at admission of the 215 patients tested for Bordetella *pertussis*

Diagnosis at admission	Patients (*n* = 215)	BP+ (*n* = 53)	Percent
Bronchiolitis	101	20	19.8
Apnea	41	8	19.5
Fever in infants	23	1	4.3
Suspected pertussis	22	16	72.7
Cough	19	8	42.1
Pneumonia	4	0	0
ALTE	5	0	0

*BP+* patients with Bordetella *pertussis* positive aspirate, *ALTE* acute life threatening episode

Out of 215 patients tested, 53 had a positive RT-PCR for BP (24.7 %). Of the 162 patients resulted negative for BP, 119 were positive for RV infections (55.3 %): RSV was diagnosed in 48 (40.3 %), Rhinovirus in 37 (31.1 %), Parainfluenzae Virus in 9 (7.6 %), Adenovirus in 4 (3.4 %), Metapneumovirus in 4 (3.4 %), Influenzae Virus in 3 (2.5 %), Coronavirus in 3 (2.5 %), Rhinovirus + Adenovirus in 4 (3.4 %), Rhinovirus + Coronavirus in 2 (1.7 %), RSV + Adenovirus in 2 (1.7 %), Parainfluenzae virus + Metapmeumovirus in 2 (1.7 %), RSV + Coronavirus in 1 (0.8 %).

No etiological agent was identified on nasopharyngeal aspirate of 43 patients (20 %). Those patients were discharged with the following diagnoses: apnea *(ICD-9 code 78609)* (21), bronchiolitis (13), laryngitis/laryngomalacia (3), unexplained fever in infants (2), sepsis (1), pneumonia (1), intraventricular septal defect (1), HHV6 encephalitis (1).

At admission, pertussis was clinical suspected in 22 patients only on the basis of the WHO definition. Sixteen of them had a positive RT-PCR for BP, while the 6 patients resulted negative to BP were discharged with diagnosis of bronchiolitis in 5 cases (2 RSV, 2 Rhinovirus and 1 Parainfluenzae Virus) and apnea in 1 case (negative nasopharyngeal aspirate). On the other hand, among the remaining 193 patients who had a different diagnosis at admission, 37 were RT-PCR positive for BP. Thus the sensitivity of clinical diagnosis at admission was 30.2 % (19.52–43.54) and the specificity 96.3 % (92.16–98.29).

The clinical and laboratory characteristics on admission were compared between BP+ patients, RV+ patients and BP-RV- patients (Table 2). Cough, paroxysmal cough, whoop, apnea, fever, rhinorrhea, white blood count, lymphocytes count, length of symptoms before admission and length of hospital stay were statistically different among the three groups.

**Table 2:** Demographic, clinical and laboratory characteristics of study patients

	BP+	VR+	BP-/VR-	p
	(*n* = 53)	(*n* = 119)	(*n* = 43)	
Age (days)^a^	56.20 ± 21.66	51.62 ± 20.59	50.16 ± 22.96	0,3
Male gender	34 (64.2 %)	66 (55.5 %)	24 (55.8 %)	0.56
Cough	45 (84.9 %)	100 (84 %)	18 (41.9 %)	<0.001
Paroxysmal cough	16 (30.2 %)	7 (5.9 %)	1 (2.3 %)	<0.001
Apnea	30 (56.6 %)	26 (21.8 %)	23 (53.5 %)	<0.001
Emesis	10 (18.9 %)	25 (21 %)	5 (11.6 %)	0.39
Whoop	4 (7.5 %)	0	2 (4.7 %)	0.015
Fever	1 (1.9 %)	39 (32.8 %)	8 (18.6 %)	<0.001
Rhinorrhea	0	24 (20.2 %)	3 (7 %)	0.001
Dyspnea	4 (7.5 %)	19 (16 %)	10 (23.3 %)	0.1
WBC (n/mm^3^)^a^	17.432 ± 9.332	11.908 ± 5.586	11.300 ± 3.731	<0.001
L (n/mm^3^)^a^	10.553 ± 6.349	5.278 ± 2.996	5.728 ± 2.442	<0.001
CRP (mg/dl)^a^	0.19 ± 0.43	0.76 ± 1.4	0.6 ± 1.1	0.046
Length of symptoms before admission (days)^a^	9.07 ± 6.68	3.13 ± 2.87	3.72 ± 4.03	<0.001
Length of hospital stay (days)^a^	8.06 ± 4.56	6.19 ± 3.00	8.76 ± 7.99	0.001
Complications				
O_2_therapy	13 (24.5 %)	33 (27.7 %)	9 (20.9 %)	0.66
Pneumonia	3	18	3	0.12
Death	0	0	0	-

*BP+* patients with Bordetella *pertussis* positive aspirate, *RV+*: patients with respiratory virus positive aspirate, *BP-RV-* patients with Bordetella *pertussis* and respiratory virus negative aspirate, *WBC* white blood count, *L* lymphocyte count, *CRP* C-reactive protein

^a^expressed as mean ± SD

When we applied the logistic regression model to explore predictive clinical manifestations and/or laboratory test for pertussis, data showed that paroxysmal cough, absence of fever, absolute lymphocyte count >10.000 n/mm^3^ and duration of symptoms before admission ≥5 days were significantly associated with pertussis compared with other diagnoses (Table 3).

**Table 3:** Association of clinical variables with laboratory confirmed pertussis

	aOR	95 % CI	p
Age	0.995	0.972;1.019	0.68
Male gender	0.406	0.133;1.238	0.11
Cough	1.607	0.413;6.254	0.49
Paroxysmal Cough	7.535	1.495;37.985	0.01
Apnea	1.095	0.285;4.209	0.89
Emesis	0.616	0.128;2.959	0.54
Whoop	3.303	0.363;30.048	0.28
Absence of Fever	15.130	1.337;171.194	0.02
Rhinorrhea	undefined	undefined	-
Dyspnea	0.902	0.169;4.827	0.90
WBC >18.000 n/mm^3^	1.289	0.196;8.487	0.79
L >10.000 n/mm^3^	21.922	3.368;142.693	0.001
CRP <0.5 mg/dl	0.598	0.149;2.402	0.46
Length of symptoms before admission >5 days	3.782	1.316;10.870	0.01

*WBC* white blood count, *L* lymphocyte count, *CRP* C-reactive protein

Notably, when we analyzed the length of symptoms before admission of patients with BP+ we found that 20 patients (37.7 %) reported symptoms for less than 7 days; 20 patients (37.7 %) reported symptoms for 7- < 14 days and 13 of them (24.5 %) reported symptoms for more than 14 days.

Therefore, our data showed that apnea is not predictive for pertussis, but it’s a frequent clinical manifestation (30/53); among BP+ patients, 22 (41.5 %) reported apnea associated with cough and cyanosis, while 8 of them (15.1 %) reported apnea alone not associated with other symptoms.

Complications (oxygen requirement and pneumonia) were not statistically different in the three groups. No deaths were reported (Table 2).

Regarding ongoing antibiotic therapy, 34 of our patients (9 BP+, 21 VR+, 4 BP-/VR-) had already started antibiotics before admission; particularly, 19 patients (7 BP+, 10 VR+, 2 BP-/VR-) had already started macrolide therapy when specimens were collected.

**Fig. 1:**
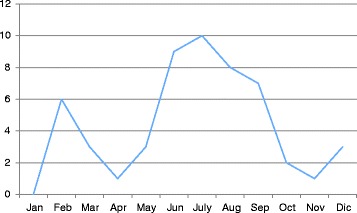
Pertussis cases by month

When we analyzed the seasonal trend of our BP+ patients, we found that the maximum incidence was between June and September, but we had cases even in winter with a peak in February (Fig. 1).

With regard to coinfection, of the 53 pertussis cases, 18 (34 %) had a positive RV result in addition to BP: 8 patients had PCR positive for Rhinovirus, 4 for Coronavirus, 1 for RSV, 1 for Metapneumovirus, 1 for Parainfluenza Virus, 1 for Influenza + Coronavirus, 2 for Rhinovirus + Parainfluenza. We didn’t find any significant differences between patients with pertussis as monoinfection and patients with pertussis plus RV infection.

## Discussion

The clinical suspicion of pertussis is not easy in infants. Among the 53 patients BP+, pertussis was suspected only in 16 patients (30.2 %) at the admission. Clinical suspicion has a low sensitivity in this age group. In absence of a systematic use of laboratory tests for the diagnosis of pertussis in infants with respiratory symptoms, many infants with BP infection may go unrecognized [17]. In a hospital environment, the lack of recognition of such an infection may represent a severe risk for hospital outbreaks, since pertussis may be transmitted to contacts if appropriate antibiotic therapy is not applied. As a matter of fact we did not observe any hospital secondary pertussis case over the study period.

Our data suggest that, despite routine surveillance shows a low incidence of pertussis in Italy, BP infection is still circulating in unvaccinated infants and nearly 25 % of patients younger than 3 months of age hospitalized with respiratory symptoms resulted positive for BP in our case series, according to what previously reported [18]. Clinical manifestations of pertussis can overlap with those of other diseases and can be atypical. Nonetheless our data, in line with the literature [19–21], suggest that some feature should alert clinicians to suspect pertussis: the hallmark clinical characteristic is paroxysmal cough; fever is usually absent and laboratory findings showed marked lymphocytosis. On the other hand the absence of typical symptoms does not exclude the diagnosis of pertussis.

Furthermore, it has been demonstrated that vaccinated children and adolescents could have more silent or mild pertussis infections [22, 23]; so we might expect that infants born to mothers who had received Tdap during pregnancy could present a modified pertussis. In our series, no infants were born to mothers who had received a booster during pregnancy.

Of note, eight of our patients (15.1 %) with confirmed BP infection were admitted with diagnosis of apnea, not associated with other symptoms, according to what previously reported by Crowcroft and Shojaei [24, 25]. In the ECDC pertussis case definition of 2012, apnoeic episodes in infants are included in the clinical criteria [26].

The lack of recognition of BP infection in infants has important consequences for surveillance. The number of infections that our hospital detected in the study period does not seem consistent with national surveillance data that suggest a low circulation of pertussis in the first year of life. Although we may have experienced an increased incidence limited to our geographic area, we speculate that other health facilities that do not systematically apply laboratory confirmation to all infants with respiratory symptoms may miss cases as well.

We did not observe deaths in our case series. Since cases included in our study were admitted in a general pediatrics ward we cannot exclude that other severe cases were admitted to pediatric intensive care unit (PICU). Crowcroft et al. [24] in a previously study, described that about 20 % of infants <5 months admitted to London PICU, with respiratory failure, apnea, or acute life threatening episode had pertussis. They reported two cases of death.

One of the barriers to wide use of RT-PCR for pertussis may be costs associated with laboratory tests. Beyond the tremendous impact of early therapy in pertussis cases, it should be underlined that RT-PCR for pertussis has an additional cost of nearly 30 euros or 38 USD per test.

The use of molecular assays has become standard of care in many setting recently. Mainly PCR has the advantage of offering result within several hours and in most of the cases, it is considerated highly sensitive especially during the “late stage” of the disease. Moreover, because is not affected by the use of drugs, one of the major usefulness respect to cultures methods is that specimens could be collected after antibiotic treatment has been started. Phenotypic cultures, although highly specific, fall in sensitivity with a reported range between 30 and 60 % of BP detection and increased time for releasing test result compared with PCR [27]. We account as great limitation of PCR that it’s unable to differentiate asymptomatic carriers. In our series, all the patients were admitted for acute respiratory symptoms and were unvaccinated so we considered them as affected by pertussis and to be properly treated.

The PCR target used for diagnosis in this study was IS481, consequently we cannot distinguish between BP, Bordetella *holmesii* and Bordetella *bronchiseptica.* Therefore, we consider it is unlikely that our data are a possible overestimation of true data.

The IS481 sequence is represented from 50 up to 238 copies per cel in BP considering this an advantage in term of high sensitive detection. Notwithstanding, microbiologist, infectious disease practitioners and pediatrics should be acquainted with the low presence of this target in Bordetella *holmesii* (8 to 10 copies per genome) and Bordetella *bronchiseptica* as well [28–30]. Some laboratories perform a second PCR assay on IS481-positive specimens using either a BP- or a Bordetella *holmesii-*specific target or both. However, these other targets are at least ten fold less sensitive than IS481 target since they are present in fewer copies of the genome. A number of potential target sequences have been proposed to increase the diagnosis of BP and Bordetella *holmesii* but there is no recommendation on what is the best PCR diagnosis strategy to use at the present time [16].

We recommend referring the final species differentiation of pertussis illness depending on several factors as by the setting of patients (more often related to immunocompromised individuals), or being contingent on epidemiology contest [31].

Regarding Bordetella *parapertusiss*, in many clinical laboratories due to the high prevalence of pertussis in order to better exploit the species responsible of pertussis disease, it is suggested to investigate at the same time RT-PCR assays by using IS481 for BP and IS 1001 specific of Bordetella *parapertussis*. These double check methods could represent an appropriate procedure in order to estimate the prevalence of Bordetella *pertussis/parapertussis* infections, and to determinate their epidemiologic characteristics in infants [32].

Although the duration of symptoms in BP+ patients was longer than in the other diagnoses, 40 patients (75.5 %) of 53 BP+ showed cough for less than 14 days. According to the clinical case definition of CDC, WHO [33] and ECDC [26], none of those patients should have been reported. Similar observations were reported also by others [25, 34]. The case definitions and classifications played an important role in improving the sensitivity of pertussis diagnosis in epidemiological surveys. But if these criteria are used purely clinically to select pertussis cases for confirmation in this age group, this may result in considerable under-diagnosis of pertussis. The clinical suspicion does not always meet all the clinical criteria. Therefore, the current clinical criteria of pertussis are all based on paroxysmal cough, but as *van den Brink et al* have previously demonstrated, it is not a good predictor in atypical pertussis infections [34]. The development of an age-related case definition, as suggested by the GPI [33], may be important to better assess the real burden of pertussis in infants.

Our data support what has been previously reported by Gonfiantini et al. [10] about the pertussis seasonality. Many patients were affected by pertussis during summer period; nevertheless some cases were reported even in winter when most of respiratory viruses circulate among population.

BP and RV co-infection was documented in 34 % of our subjects. Conflicting results have been reported regarding the frequency of these concomitant [34–37]. The short duration of surveillance, the inclusion of highly selected patient groups based on age and diagnosis may have contributed to these heterogeneous findings. Therefore, in most of the previous studies, has been taken into account only the co-infection between RSV and BP. In our study we analyzed co-infections between BP and the most frequent RV.

From the clinical point of view, it’s important to recognize co-infections, both for infection control and clinical management. A diagnosis of respiratory virus infection does not exclude pertussis and viceversa.

This study has some limitations. The retrospective data collection from medical records can contain inaccuracies regarding clinical information, but objective parameters were analyzed in order to reduce the possibility of bias. Second, we cannot say if our results may be generalizable to other pediatric settings in our country. Nonetheless the use of laboratory test for pertussis confirmation is not widespread in Italy and, despite EU recommendations for case definition, most reported cases are still diagnosed on a clinical base only. Lastly nineteen of our patients were treated with macrolides before admission; this may have mitigated the clinical manifestations in some cases.

Several strategies have been proposed for prevention of pertussis in infants [38]. These include immunization of adolescents and adults with tetanus, diphtheria toxoid and acellular pertussis (Tdap) to boost waning immunity against pertussis. Recent study, however, demonstrated that after Tdap introduction in adolescents, the incidence in this age group was reduced but the average incidence of pertussis among infants younger than 1 year did not significantly change. This is probably because of increased vaccination coverage of those patients at the highest risk to transmit disease is needed before the indirect effects of Tdap are fully realized [39, 40].

Another proposed strategy is maternal immunization during the third trimester of pregnancy; from October 2011, the Advisory Committee on Immunization Practices (ACIP) recommended that unvaccinated pregnant women receive a dose of Tdap [41]. Vaccination of women with Tdap during pregnancy is expected to provide protection to infants from pertussis until they are old enough to be vaccinated themselves [42]. Tdap given to pregnant women stimulate the development of maternal antipertussis antibodies, which pass through the placenta, likely providing the newborn with protection against pertussis in early life, and protect the mother from pertussis around the time of delivery, making her less likely to become infected and transmit pertussis to her infant. In England an immunization program against pertussis for pregnant women was introduced in October 2012 in response to a marked increase in pertussis cases, particularly in young infants. The program achieved 60 % vaccine coverage and 90 % vaccine effectiveness in preventing infant disease was demonstrated [43].

Vaccination of pregnant women is considered likely to be the most cost-effective complementary strategy to prevent pertussis-associated infant mortality [44].

Moreover, postpartum maternal immunization with Tdap, as well as immunization of close household members to prevent transmission of infection to vulnerable infants (cocooning strategy) is currently recommended by the Advisory Committee for Immunization Practices to the Center of Disease Control and Prevention [45]. Since the impact of these strategies cannot be accurately monitored without reliable surveillance data, we believe that considering *Bordetella pertussis* infections in infants ≤3 months of age hospitalized with respiratory symptoms is essential.

## Conclusions

In conclusions, clinical manifestations of pertussis in infants can overlap with several different diseases. Sometimes presentation may mimic a viral respiratory tract infection. Our data support a routinely use of RT-PCR for pertussis in all infants ≤3 months of age with any respiratory symptoms in order to implement appropriate control measures in hospital and in the community at large as the clinical suspicion is often not enough to well recognize pertussis infection.

## Abbreviations

BP, Bordetella *pertussis* ; RV, respiratory viruses; RSV, respiratory syncytial virus; IC, internal control; BP+, Bordetella *pertussis* positive aspirate; RV+, respiratory viruses positive aspirate; BP-RV-, Bordetella *pertussis* and respiratory viruses negative aspirate; RT-PCR, real time polymerase chain reaction; PICU, pediatric intensive care unit; ACIP, advisory committee on immunization practices
